# *In vitro* substrate phosphorylation by Ca^2+^/calmodulin-dependent protein kinase kinase using guanosine-5^′^-triphosphate as a phosphate donor

**DOI:** 10.1186/1471-2091-13-27

**Published:** 2012-12-05

**Authors:** Saki Yurimoto, Tomohito Fujimoto, Masaki Magari, Naoki Kanayama, Ryoji Kobayashi, Hiroshi Tokumitsu

**Affiliations:** 1 Department of Signal Transduction Sciences, Faculty of Medicine, Kagawa University, 1750-1 Miki-cho, Kita-gun, Kagawa, 761-0793, Japan; 2 Department of Bioscience and Biotechnology, Graduate School of Natural Science and Technology, Okayama University, 3-1-1, Tsushima-naka, Kita-ku, Okayama, 700-8530, Japan

**Keywords:** Calmodulin, CaMKK, Phosphate donor, GTP, Phosphorylation

## Abstract

**Background:**

Ca^2+^/calmodulin-dependent protein kinase kinase (CaMKK) phosphorylates and activates particular downstream protein kinases — including CaMKI, CaMKIV, and AMPK— to stimulate multiple Ca^2+^-signal transduction pathways. To identify previously unidentified CaMKK substrates, we used various nucleotides as phosphate donors to develop and characterize an *in vitro* phosphorylation assay for CaMKK.

**Results:**

Here, we found that the recombinant CaMKK isoforms were capable of utilizing Mg-GTP as a phosphate donor to phosphorylate the Thr residue in the activation-loop of CaMKIα (Thr^177^) and of AMPK (Thr^172^) *in vitro*. Kinetic analysis indicated that the *K*_m_ values of CaMKK isoforms for GTP (400-500 μM) were significantly higher than those for ATP (~15 μM), and a 2- to 4-fold decrease in *V*_max_ was observed with GTP. We also confirmed that an ATP competitive CaMKK inhibitor, STO-609, also competes with GTP to inhibit the activities of CaMKK isoforms. In addition, to detect enhanced CaMKI phosphorylation in brain extracts with Mg-GTP and recombinant CaMKKs, we found potential CaMKK substrates of ~45 kDa and ~35 kDa whose Ca^2+^/CaM-induced phosphorylation was inhibited by STO-609.

**Conclusions:**

These results indicated that screens that use STO-609 as a CaMKK inhibitor and Mg-GTP as a CaMKK-dependent phosphate donor might be useful to identify previously unidentified downstream target substrates of CaMKK.

## Background

Ca^2+^/calmodulin-dependent protein kinase kinase (CaMKK) has been classified as a novel member of the calmodulin kinase (CaMK) family that specifically phosphorylates a single Thr residue (Thr^177^ or Thr^196^, respectively) within the activation loop in each of two multifunctional calmodulin (CaM) kinases, CaMKI and CaMKIV; these phosphorylation events cause a large increase in catalytic efficiency [1-3]. Accumulated biochemical evidence indicates that CaMKK phosphorylates Akt/Protein kinase B [4] and AMPK (AMP-activated protein kinase) family members including the catalytic subunit of AMPK (AMPKα) at Thr^172^[5-8] and SAD-B (known as a brain-specific kinase, BRSK1) at Thr^189^[9]; either phosphorylation event causes significant catalytic activation, and these findings indicate that CaMKK confers Ca^2+^ dependence on other signalling pathways. In mammals, two CaMKK genes (CaMKKα and CaMKKβ) have been identified, and both are highly expressed in the brain; the α isoform is also expressed in various peripheral tissues such as thymus and spleen [10]. A CaMKK gene has been found in *Caenorhabditis elegans* and in *Aspergillus nidulans*, and the proteins encoded by these genes are components of the CaMK cascade within the respective organisms [11,12]. Interestingly, both mammalian CaMKK isoforms bind to Ca^2+^/CaM as well as to CaMKI and CaMKIV proteins that function downstream of Ca^2+^/CaM complexes [13,14]. Indeed, Ca^2+^/CaM binding is absolutely required for the relief of CaMKKα autoinhibition [15], which results in its activation like other CaMKs. Previous structural and functional studies of CaMKK have revealed a novel CaM-binding motif (1-16) [16], and the unique feature of the CaM-binding segment in CaMKK is required for the autoinhibitiory mechanism through Ile^441^ in CaMKKα [15].

Many cell types depend on a functional CaM-kinase cascade that leads to activation of CaMKI and CaMKIV in response to Ca^2+^ mobilization. The CaMKK/CaMKIV cascade has an important role in Ca^2+^-dependent regulation of gene expression that is mediated by phosphorylation of transcription factors such as cAMP-response element binding protein (CREB) [17]. Analysis of CaMKIV-deficient mice revealed that the CaMKIV-mediated pathway plays an important role in the development and function of the cerebellum and is critical for both male and female fertility [18,19]. The CaMKK/CaMKI cascade has been shown to be involved in various neuronal functions, including spinogenesis [20], dendritic arborization [21] and cortical axon elongation [22]. Recent accumulated data have shown that Ca^2+^-dependent phosphorylation and consequent activation of AMPK is mediated by CaMKKβ when T-cells are activated via the antigen receptor [23] or when HeLa cells were treated with a Ca^2+^ ionophore [24]. Based on these results, CaMKK is predicted to act as a regulatory protein kinase in various Ca^2+^-dependent cellular processes *in vivo*. Therefore, identification of novel target(s) of CaMKK is important for the clarification of the CaMKK-mediated signaling pathway. Here, we found and characterized an enzymatic feature of CaMKK isoforms that might be useful in screens for novel targets of CaMKK; specifically, these enzymes can use GTP, as well as ATP, as a phosphate donor.

## Results and discussion

### CaMKK is capable of using Mg-GTP as a phosphate donor

**Figure 1:**
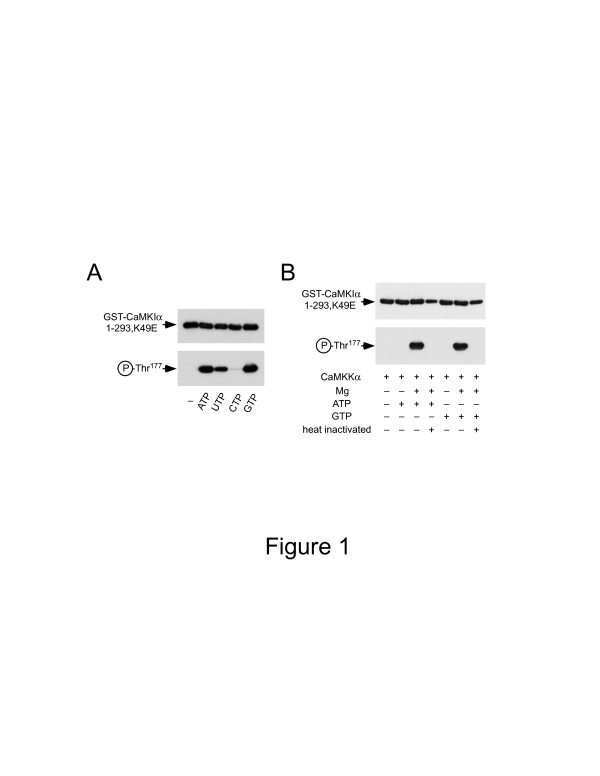
**CaMKK can utilize various nucleotides as phosphate donors *in vitro. A***. Purified recombinant CaMKKα was incubated with GST-CaMKIα (1-293, K49E, 0.5 mg/ml) at 30°C for 10 min in the solution containing 50 mM HEPES (pH 7.5), 1 mM DTT, 1 mM CaCl_2_, 10 mM Mg(CH_3_COO)_2_, and 10 μM CaM in the absence (-) or presence of either 1 mM ATP, UTP, CTP, or GTP. After terminating the reaction, samples were subjected to western blot analysis with either anti-GST antibody (***A****, upper panel*) or anti-phospho-CaMKI antibody (***A****, lower panel*). Similar results were obtained for at least three independent experiments. ***B***. Purified recombinant CaMKKα (+) was incubated with GST-CaMKIα (1-293, K49E, 0.5 mg/ml) at 30°C for 10 min in a solution used in panel A with (+) or without (-) 10 mM Mg(CH_3_COO)_2_, in the absence (-) or presence (+) of 1 mM ATP or 1 mM GTP. The reaction mixtures were heated at 68°C for 10 min (*heat inactivated* +) or kept on ice (-) before initiating the phosphorylation reaction. After each reaction was terminated, samples were subjected to western blot analysis with either anti-GST antibody (***B***, *upper panel*) or anti-phospho-CaMKI antibody (***B***, *lower panel*).

In general, *in vitro* phosphate labeling of substrates in crude tissue or cell lysates is an essential experimental step in the identification of novel downstream targets for protein kinases; nevertheless, it is technically difficult to phosphorylate physiologically relevant substrates via target protein kinases in crude tissue and cell extracts. If ATP is used as a phosphate donor in phosphorylation reaction that use crude tissue or cell extracts as the kinase source, background phosphorylation by myriad endogenous protein kinases is unavoidable since ATP is utilized by every known protein kinase as a phosphate donor. To identify novel substrates for CaMKK, we first tested whether CaMKK can utilize other nucleotides as substitutes for ATP in the phosphorylation reaction. To examine the ability of recombinant CaMKKα isoform to use non-ATP nucleotides *in vitro* (Figure 1A), we used 1 mM GTP, UTP, or CTP, as well as 1 mM ATP (the positive control), as the sole phosphate donor, CaMKK as the kinase, and GST-CaMKIα (1-293, K49E; a kinase dead mutant lacking the Ca^2+^/CaM-binding region) as the substrate in Thr^177^ phosphorylation reactions; all reactions included Mg^2+^. We used this CaMKI mutant as a CaMKK substrate because it does not need to bind Ca^2+^/CaM to be phosphorylated by CaMKK; additionally, this mutant allows us to rule out any confounding effects of CaMK-mediated feedback phosphorylation of CaMKK [15]. Western blot analysis and an anti-phosphoThr^177^ monoclonal antibody were used to detect site-specific phosphate incorporation into GST-CaMKIα 1-293, K49E. CaMKKα could use GTP, UTP, or the positive control (ATP) as a phosphate donor (Figure 1A); however, phosphorylation with UTP was less efficiently than that with GTP or ATP; phosphate incorporation with UTP was ~50% of the maximum incorporation observed within 10 min with ATP or GTP. Moreover, CaMKKα was incapable of using CTP as a phosphate donor. Next, we used GTP, as well as ATP, *in vitro* under various conditions to confirm the kinase activity of recombinant CaMKKα (Figure 1B). The CaMKKα isoform was capable of phosphorylating Thr^177^ in the CaMKI substrate with GTP only in the presence of Mg^2+^ as well as Mg-ATP. When we incubated the reaction mixture at 68°C for 10 min before initiating the phosphorylation reaction, no phosphate incorporation into Thr^177^ of the CaMKI mutant was observed. This result indicated that this was an enzyme-catalyzed reaction because conditions designed to denature enzymes abolished the observed activity.

### Isoform specificity of CaMKK for utilizing Mg-GTP

**Figure 2:**
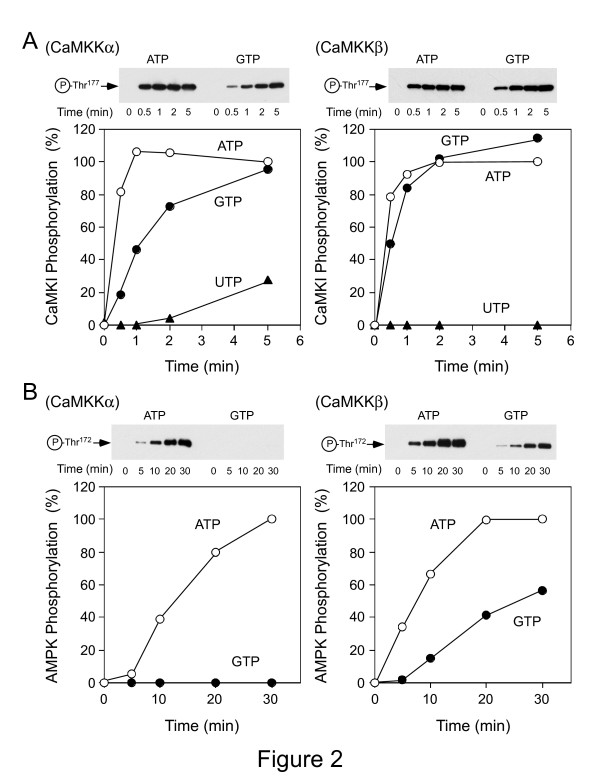
**Time course of CaMKK-mediated phosphorylation with various nucleotides of GST-CaMKIα (1-293, K49E) or AMPKα.** Purified recombinant CaMKKα (*left panels*) or CaMKKβ (*right panels*) was incubated with either GST-CaMKIα (1-293, K49E, ***A***) or AMPK (***B***) at 30°C for the indicated time periods in a solution containing 50 mM HEPES (pH 7.5), 10 mM Mg(CH_3_COO)_2_, 1 mM DTT, 1 mM CaCl_2_, 10 μM CaM**,** in the presence of either 1 mM ATP (*•*), 1 mM GTP (·) or 1 mM UTP (▴) as described in "Methods." After each reaction was terminated, samples were subjected to western blot analysis with either anti-phospho-CaMKI antibody (***A****, upper images*) or anti-phospho-AMPKα antibody (***B****, upper images*) followed by quantitating the immunoreactive bands by densitometric scanning of the scientific imaging film used for detection of chemiluminescence. Phosphate incorporation into each CaMKK target is expressed as a percentage of the value of the reaction for the 5-min time point (30-min time point for AMPK) in the presence of ATP. Results are representative of at least two independent experiments.

CaMKK is composed of either α or β isoforms [10,11] that phosphorylate and activate AMPK as a native substrate in addition to CaMKI. Next, we characterized the ability of the CaMKK isoforms to phosphorylate these two downstream substrates with 1 mM ATP, GTP, or UTP in the presence of 10 mM Mg(CH_3_COO)_2_ (Figure 2). Under the conditions of our time course experiments examining phosphorylation, both CaMKK isoforms were able to use ATP or GTP to phosphorylate Thr^177^ in CaMKIα 1-293, K49E, and the kinetics of the reactions were comparable (Figure 2A). Consistent with the results of Figure 1A, CaMKKα could utilize Mg-UTP, but with slower catalysis; moreover, the β isoform was incapable of using UTP under these experimental conditions. On the other hand, only CaMKKβ was capable of phosphorylating the α subunit of AMPK at Thr^172^ with Mg-GTP, while both CaMKK isoforms could phosphorylate AMPK with Mg-ATP (Figure 2B). This finding was consistent with the observation that the GST-fused catalytic domain of CaMKKβ (residues 162-470), but not CaMKKα catalytic domain (residues 126-434), phosphorylated AMPK with Mg-GTP (data not shown). Neither CaMKK isoforms could use UTP or CTP to phosphorylate AMPK (data not shown). These results indicated that CaMKK isoforms could use Mg-GTP for target phosphorylation depending on downstream substrates.

### Kinetic analysis of CaMKK isoforms

To further characterize the ability of CaMKK isoforms to use GTP as a phosphate donor, we then compared the kinetic parameters of CaMKK isoforms with ATP or GTP as the phosphate donor and GST-CaMKIα 1-293, K49E as the CaMKK substrate. The *K*_m_ values of both CaMKK isoforms for GTP were ~30-fold higher (400~500 μM) than those for ATP (~15 μM); however, a 2- to 4-fold decrease in *V*_max_ was observed with GTP for both CaMKK isoforms under our experimental conditions (Table 1). The *V*_*max*_*/K*_*m*_ values of GTP for both CaMKK isoforms were two order of magnitude lower than those of ATP (Table 1). Thus, GTP is considered a less efficient substrate for CaMKKs than ATP.

**Table 1:** Kinetic Parameters of CaMKK Isoforms

**CaMKK**	**Phosphate**	***K***_**m**_	***V***_**max**_	***V***_**max**_***/ K***_**m**_
**isoforms**	**Donor**	**(μM)**	**(nmol/min/mg)**	
CaMKKα	ATP	15	625	41.7
	GTP	444	312	0.7
CaMKKβ	ATP	15	286	19.1
	GTP	507	77	0.2

CaMKK activities were measured using GST-CaMKIα (1-293, K49E) as a substrate at 30°C in the presence of one of several concentrations (50 – 400 μM) of either ATP or GTP as described in “Methods”.

### Inhibition of CaMKK activities using Mg-GTP by STO-609

**Figure 3:**
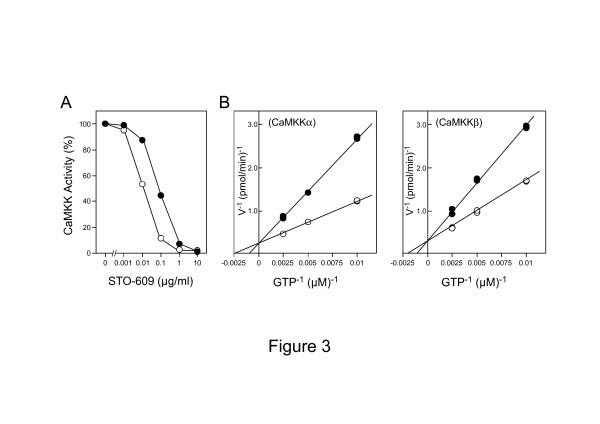
**Effect of STO-609 on the activities of CaMKK isoforms with Mg-GTP. *A***. Recombinant CaMKKα (*·*) or recombinant CaMKKβ (*○*) was incubated with GST-CaMKIα (1-293, K49E, 0.5 mg/ml) at 30°C for 4 min in the presence of 50 μM [γ-^32^P]GTP and 2 mM CaCl_2_/4 μM CaM with various concentrations of STO-609 (0 – 10 μg/ml) as described in "Methods." Activities are expressed as a percentage of the value in the absence of STO-609. Results represent mean and S.E. of three experiments. ***B***. Protein kinase activities of purified recombinant CaMKKα (***B***, *left panel*) and β (***B****, right panel*) were measured with various concentrations (100 – 400 μM) of [γ-^32^P]GTP in either the absence (*○*) or presence (*·*) of 0.1 μg/ml STO-609 for CaMKKα and in either the absence (*○*) or presence (*·*) of 0.01 μg/ml STO-609 for CaMKKβ. The results represent duplicate experiments and are presented as double reciprocal plots (*Lineweaver-Burk*).

Based on the enzymatic characterization of CaMKK isoforms with regard to the ability to utilize various nucleotides as described above, Mg-GTP can be used for phosphate-labeling of CaMKK substrates in crude tissue extracts. However, a number of other protein kinases — including casein kinase II, CaMKII and protein kinase C — can use Mg-GTP as a phosphate donor [25-28]. Therefore, we need an additional selection method to identify CaMKK-specific substrates that are phosphorylated by CaMKK isoforms using Mg-GTP. Previously, we synthesized 7*H*-benzimidazo[2,1-a]benz[de]isoquinoline-7-one-3-carboxylic acid (STO-609) as a CaMKK inhibitor that has been shown to be an ATP-competitive compound [29]. Next, we tested whether STO-609 was capable of inhibiting the activity of each CaMKK isoform when 50 μM [γ-^32^P]GTP was used as a phosphate donor. CaMKKα activity was inhibited by > 90% in presence of 1 μg/ml STO-609, and CaMKKβ was inhibited by > 90% in presence of 0.1 μg/ml STO-609 (Figure 3A). CaMKKβ was ~7 times more sensitive to STO-609 than was CaMKKα when Mg-GTP was used as the phosphate donor (CaMKKα, IC_50_ = 80 ng/ml; CaMKKβ, IC_50_ = 12 ng/ml); this inhibitory profile was similar to that with 50 μM Mg-ATP (CaMKKα, IC_50_ = 120 ng/ml; CaMKKβ, IC_50_ = 40 ng/ml) as the phosphate donor [29]. Kinetic analysis of the inhibition of CaMKK isoforms by STO-609 (Figure 3B) showed the degree of inhibition observed with varying concentrations of GTP (100 – 400 μM) in the presence or absence of STO-609 (0.1 μg/ml) for CaMKKα (Figure 3B*left panel*) and in the presence or absence of STO-609 (0.01 μg/ml) for CaMKKβ (Figure 3B*right panel*). As there was no change in the *V*_max_ value for the two CaMKK isoforms, the apparent *K*_m_ value for GTP increased with increasing concentrations of STO-609, indicating that the inhibition was competitive with respect to GTP.

### Detection of phosphorylation of CaMKK targets in rat brain extracts

**Figure 4:**
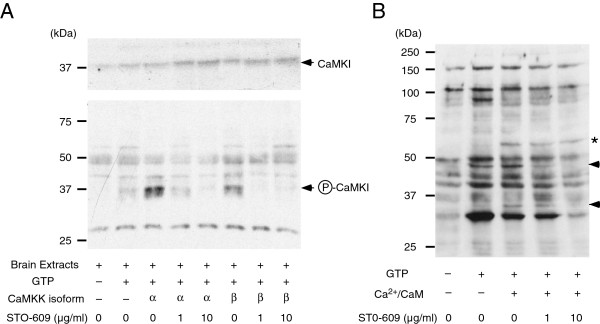
***In vitro *phosphorylation of CaMKK substrates in rat brain extract with Mg-GTP. *A***. Rat brain extract (5.6 mg/ml) was incubated without (-) or with recombinant CaMKKα (α) or β (β) isoform (2 μg) in the absence (-) or presence (+) of 0.1 mM GTP at 30°C for 1 min in a solution containing 50 mM HEPES (pH 7.5), 10 mM Mg(CH_3_COO)_2_, 2 mM DTT, 2 mM CaCl_2_ and 4.5 μM CaM and indicated concentrations (0, 1, and 10 μg/ml) of STO-609, followed by western blot analysis with anti-phospho-CaMKI antibody. ***B***. Rat brain extract (6.6 mg/ml) was incubated without (-) or with 1 mM GTP (+) at 30°C for 30 min in the presence of either 2 mM EGTA (-) or 2 mM CaCl_2_/11 μM CaM (+) in a solution containing 50 mM HEPES (pH 7.5), 10 mM Mg(CH_3_COO)_2_, 2 mM DTT and indicated concentrations (0, 1, or 10 μg/ml) of STO-609, followed by western blot analysis with anti-phospho-threonine antibody. The *asterisk* and *arrowheads* indicate rat brain proteins whose phosphorylation was induced by Ca^2+^/CaM. Results are representative of at least three independent experiments.

Finally, we attempted to detect the phosphorylated substrates of CaMKK within tissue extracts that were incubated with recombinant CaMKKs in the presence of Mg-GTP. When we used rat brain extracts, the phosphorylation of CaMKI at the activation Thr residue (Thr^177^ in CaMKIα isoform) was enhanced by incubation of both CaMKK isoforms, and this phosphorylation of CaMKI by CaMKK isoforms was completely inhibited by STO-609 (Figure 4A). In this experiment, we used 100 μM of GTP that is relatively lower than *K*_m_ value for GTP (CaMKKα, 444 μM; CaMKKβ, 507 μM, Table 1) in order to reduce the background phosphorylation. These results indicated that CaMKK was capable of using Mg-GTP as a phosphate donor to phosphorylate the target substrate even in the crude tissue lysate. We then used western blot analysis and an anti-phospho-threonine antibody to examine the phosphorylation profile of rat brain extract that was incubated with Mg-GTP in the presence of either EGTA or the CaMKK activator, Ca^2+^/CaM (Figure 4B). In this experiment, we didn’t add recombinant CaMKKs into the rat brain extract to minimize the kinase concentration in the phosphorylation reaction but the endogenous CaMKK activity should be apparently enhanced by using a relatively high concentration of GTP (1mM) as compared to the *K*_m_ values for GTP. Although a number of proteins in brain extracts had already been phosphorylated at Thr residues, we detected endogenous rat brain proteins with molecular weight of ~60 kDa, ~45 kDa and ~35 kDa (Figure 4B, indicated by an *asterisk* and *arrow heads*) that exhibited increased phosphorylation levels following the addition of Ca^2+^/CaM. However, phosphorylation of a ~60 kDa protein was not suppressed by even high concentration (10 μg/ml) of STO-609; this finding suggested that the 60 kDa phosphoprotein was not being phosphorylated by CaMKK. In addition, induced phosphorylation of the 45 kDa protein by GTP was observed in the absence of Ca^2+^/CaM. This is probably due to phosphorylation by CaMKKβ whose kinase activity is highly Ca^2+^/CaM-independent [30]. The phosphorylation levels of a ~45 kDa and a ~35 kDa protein were significantly reduced by addition of STO-609, indicating that these phosphoproteins might be substrates of CaMKK (Figure 4B). It is noteworthy that we couldn’t clearly detect the phospho-45 kDa and -35 kDa proteins when we used Mg-ATP as a phosphate donor because of enhanced background phosphorylation (data not shown).

## Conclusions

Searching for target substrates is always important to evaluate the physiological significance of a protein kinase. Since 1995, when CaMKK was cloned as an activator for CaMKI and CaMKIV [10], we have attempted to develop methods using enzyme-substrate interactions and an ATP-analogue to search for novel CaMKK targets; in that time, we found two potential CaMKK targets — SAD-B [9] and Syndapin 1 [31]. Here, we attempted to examine various nucleotides as phosphate donors for *in vitro* phosphorylation of target substrates by CaMKKs. We have shown that CaMKK isoforms were capable of using Mg-GTP as a phosphate donor. Usage of this nucleotide for CaMKK activity varies depending on downstream targets. 1) Both CaMKK isoforms were capable of phosphorylating CaMKIα (at Thr^177^) with Mg-GTP, as well as with Mg-ATP. 2) AMPKα can be phosphorylated (at Thr^172^) with Mg-GTP only by CaMKKβ, not by CaMKKα. Although very few protein kinases are known to use GTP as well as ATP, CaMKK is not the first protein kinase to use both nucleotides. Casein kinase II has been well characterized in its ability to use GTP and ATP [26,28,32]. Very recently, *Drosophila* and rat CaMKIIα have been shown to utilize GTP for exogenous substrate phosphorylation and autophosphorylation *in vitro*[25]. In addition, previous reports have shown that four mammalian serine/threonine protein kinases — including protein kinase Cδ, Nercc1, mst-3 and AGT (*O*^6^-alkylguanine-DNA alkyltransferase) kinase — are able to use GTP as a substrate [27,33-35]. However, the physiological significance of GTP-dependent phosphorylation has been unexplored because ATP has been recognized as the only biologically relevant phosphate donor for protein kinases. *In vitro*, we found that the phosphorylation of a CaMKK target protein, specifically CaMKI, was induced by incubation of rat brain extract with recombinant CaMKKs in the presence of Mg-GTP and Ca^2+^/CaM, and that this phosphorylation was inhibited by STO-609, a CaMKK inhibitor. Based on these results, we suggest that this enzymatic feature of CaMKKs, specifically the ability to use GTP or UTP in place of ATP as phosphate donors *in vitro*, might be useful in screens for novel CaMKK targets. However, careful consideration should be required to use Mg-GTP as a phosphate donor for CaMKK isoforms, since the differential effect of Mg-GTP on the activities of CaMKK isoforms (Figure 2B). Furthermore, the specificity of the CaMKK-mediated phosphorylation reaction could be confirmed by addition of the CaMKK inhibitor, STO-609, even though some endogenous protein kinases have been shown to be capable of using GTP as a phosphate donor *in vitro*. Indeed, we detected two potential CaMKK target proteins with molecular weight of ~45 kDa and ~35 kDa whose phosphorylation was induced by incubation of rat brain extract with Mg-GTP and Ca^2+^/CaM and was inhibited by STO-609. Based on the molecular weight of those phosphoproteins on SDS-PAGE, ~35 kDa phosphoprotein might be a member of CaMKI isoforms (Figure 4A). However, among various known CaMKK target kinases including CaMKI, CaMKIV, PKB, AMPK, and SAD-B [1-9], CaMKK target with a molecular weight of ~45 kDa on SDS-PAGE has not been identified. Further study to identify these putative CaMKK substrates is absolutely required to evaluate novel CaMKK-mediated signaling pathway. According to our study, *in vitro* phosphorylation assays using GTP in combination with STO-609 are expected to be a useful method for detecting CaMKK substrates and assessing its function(s) in various tissue and cells.

## Methods

### Materials

Recombinant CaMKKα and β were expressed in and purified from *Escherichia coli* as described previously [30]. Recombinant rat CaM was expressed in *E. coli* strain BL-21 (DE3) using the pET-CaM plasmid (kindly provided by Dr. Nobuhiro Hayashi, Fujita Health University, Toyoake, Japan) and then purified by phenyl-Sepharose column chromatography [36]. Mutant recombinant rat CaMKIα (1-293, K49E) was expressed in *E. coli* strain JM-109 as a GST-fusion protein and purified by glutathione Sepharose column chromatography [15]. Recombinant AMPK was expressed in *E. coli* strain BL21-CodonPlus (DE3) (Stratagene, La Jolla, CA) using the tricistronic pγ1β1His-α1 plasmid (kindly provided by Dr. Dietbert Neumann, Swiss Federal Institute of Technology, Zurich, Switzerland) and purified by Ni-NTA agarose column chromatography (Qiagen, Hilden, Germany) [37]. Rabbit IgG antibodies against AMPKα and those against phospho-AMPKα at Thr^172^ were purchased from Cell Signaling Technology, Inc. (Danvers, MA). An anti-CaMKI antibody was purchased from Santa Cruz Biotechnology, Inc. (Santa Cruz, CA). An anti-GST antibody and an anti-phospho-threonine antibody were purchased from GE Healthcare UK Ltd. (Buckinghamshire, UK) and from Invitrogen (Carlsbad, CA), respectively. An anti-phospho-CaMKI (phospho-Thr^177^) monoclonal antibody was generated as described previously [38]. STO-609 was synthesized as described previously [29]. ATP, GTP, UTP and CTP were purchased from Roche Applied Science (Indianapolis, IN).

### *In vitro* assay for CaMKK activity

Purified recombinant CaMKKs (CaMKKα, 0.9 μg/ml; CaMKKβ, 1.7 μg/ml) were incubated individually with GST-CaMKIα (1-293, K49E, 0.5 mg/ml) or AMPK (0.5 mg/ml) at 30°C for one of several defined time periods in a solution containing 50 mM HEPES (pH 7.5), 10 mM Mg(CH_3_COO)_2_, 1 mM DTT, 1 mM CaCl_2_ (2 mM in Figure 3), and 10 μM CaM (4 μM in Figure 3) in the presence of either 1 mM nucleotide or one of several defined concentrations between 50 and 400 μM of either [γ-^32^P]ATP (1,200 – 10,000 cpm/pmol) or [γ-^32^P]GTP (800 – 7,000 cpm/pmol); one of several defined concentrations of STO-609 (0-10 μg/ml in dimethyl sulfoxide at a final concentration of 4%) was included in individual reactions. Each reaction was initiated by the addition of cold nucleotide or [γ-^32^P]ATP or [γ-^32^P]GTP and terminated by addition of an equal volume of 2 x SDS-PAGE sample buffer; each terminated reaction was then subjected to SDS-PAGE or to spotting of aliquots (20 μl) onto phosphocellulose paper (Whatman P-81). These spotted phosphocellulose papers were then washed several times with 75 mM phosphoric acid. Phosphate incorporation into GST-CaMKIα (1-293, K49E) was determined using western blots generated with the gels and an anti-phospho-CaMKI antibody or using the spotted filters that were subjected to liquid scintillation counts.

### Phosphorylation within rat brain extract

Rat brain samples were homogenized with 5 volumes of homogenization buffer (150 mM NaCl, 50 mM Tris-HCl (pH 7.5), 1 mM DTT, 1 mM EGTA, 1 mM EDTA, 0.2 mM phenylmethylsulfonyl fluoride, 10 μg/ml leupeptin, 10 μg/ml trypsin inhibitor, 1% NP-40); homogenates were then centrifuged at 30,190 x g at 4°C for 30 min. The supernatant was stored at -80°C until use. Rat brain extract was incubated in a solution (100 μl) containing 50 mM HEPES (pH 7.5), 10 mM Mg(CH_3_COO)_2_, 2 mM DTT, 2 mM CaCl_2_, and CaM, 0.5 μM okadaic acid; each sample was incubated in the absence or presence of either 0.1 mM or 1 mM GTP at 30°C for 1 or 30 min with or without recombinant CaMKKs for indicated time periods. Each reaction was terminated by addition of 20 μl of SDS-PAGE sample buffer followed by western blot analysis with either anti-phospho-CaMKI antibody and anti-CaMKI antibody or anti-phospho-threonine antibody.

### Others

Staining of western blots was performed with horseradish peroxidase-conjugated anti-mouse IgG, anti-rabbit IgG (GE Healthcare UK Ltd., Buckinghamshire, UK), or anti-goat IgG antibody (Sigma, Saint Louis, MO) as a secondary antibody and chemiluminescence reagent (PerkinElmer Life Sciences, Waltham, MA) for signal detection. The intensity of the immunoreactive band was measured by densitometric scanning of scientific imaging film (KODAK BioMax Light Film, Carestream Health, Inc., Rochester, NY) for detection and measurement of chemiluminescence. Bradford reagent (Bio-Rad Laboratories, Inc., Hercules, CA) was used to estimate protein concentration; bovine serum albumin was used as the protein standard.

## Abbreviations

CaM: Calmodulin; CaMK: Ca^2+^/CaM-dependent protein kinase; CaMKK: Ca^2+^/CaM-dependent protein kinase kinase; AMPK: 5′AMP-activated protein kinase; STO-609: 7*H*-benzimidazo[2,1-a]benz[de]isoquinoline-7-one-3-carboxylic acid; GST: Glutathione S-transferase; DTT: Dithiothreitol.

## Competing interests

The authors declare that they have no competing interests.

## Authors’ contributions

SY participated in the experimental design, performance of research, data analysis and writing of the paper. TF originally started this work and participated in the kinetic analysis of CaMKKs. MM and NK participated in study design and took part in manuscript writing. RK supervised the work. HT is the principle investigator of this project and participated in research design, data analysis and writing of paper. All authors read and approved the final manuscript.
